# Planktonic sea urchin larvae change their swimming direction in response to strong photoirradiation

**DOI:** 10.1371/journal.pgen.1010033

**Published:** 2022-02-10

**Authors:** Shunsuke Yaguchi, Yuri Taniguchi, Haruka Suzuki, Mai Kamata, Junko Yaguchi

**Affiliations:** 1 Shimoda Marine Research Center, University of Tsukuba, Shimoda, Japan; 2 PRESTO, JST, Kawaguchi, Japan; New York University, UNITED STATES

## Abstract

To survive, organisms need to precisely respond to various environmental factors, such as light and gravity. Among these, light is so important for most life on Earth that light-response systems have become extraordinarily developed during evolution, especially in multicellular animals. A combination of photoreceptors, nervous system components, and effectors allows these animals to respond to light stimuli. In most macroscopic animals, muscles function as effectors responding to light, and in some microscopic aquatic animals, cilia play a role. It is likely that the cilia-based response was the first to develop and that it has been substituted by the muscle-based response along with increases in body size. However, although the function of muscle appears prominent, it is poorly understood whether ciliary responses to light are present and/or functional, especially in deuterostomes, because it is possible that these responses are too subtle to be observed, unlike muscle responses. Here, we show that planktonic sea urchin larvae reverse their swimming direction due to the inhibitory effect of light on the cholinergic neuron signaling>forward swimming pathway. We found that strong photoirradiation of larvae that stay on the surface of seawater immediately drives the larvae away from the surface due to backward swimming. When Opsin2, which is expressed in mesenchymal cells in larval arms, is knocked down, the larvae do not show backward swimming under photoirradiation. Although Opsin2-expressing cells are not neuronal cells, immunohistochemical analysis revealed that they directly attach to cholinergic neurons, which are thought to regulate forward swimming. These data indicate that light, through Opsin2, inhibits the activity of cholinergic signaling, which normally promotes larval forward swimming, and that the light-dependent ciliary response is present in deuterostomes. These findings shed light on how light-responsive tissues/organelles have been conserved and diversified during evolution.

## Author summary

The importance of light for organisms on Earth has led to the extraordinary development of sophisticated light-response systems during evolution. It is likely that light-dependent ciliary responses were initially acquired in unicellular and small multicellular organisms, but the pathway is poorly understood in deuterostomes, whose behavior mostly depends on responses involving muscle. Therefore, it is unclear whether ciliary responses to light are present and/or functional in deuterostomes since these responses may be too subtle for observation, unlike muscle responses. This raises the questions of how light-response systems were established and how they diversified during deuterostome evolution. Here, we provide clear evidence that planktonic larvae of sea urchin species, which belong to the deuterostome group, display backward swimming when light inhibits cholinergic signal-dependent forward swimming.

## Introduction

It is essential for organisms to precisely respond to extrinsic signals, such as light, gravity, and temperature. Among these signals, light is very important for most organisms on Earth because it provides energy, visual information, and cues for circadian rhythms. Therefore, motile organisms have developed photoreception, motor organs/organelles, and signaling systems that interact to promote positive or negative phototaxis/reflexes. For example, even single-celled organisms, such as Chlamydomonas, change the beating pattern of cilia/flagella when photoirradiated [1]. As another example, another group of nonmetazoans, the choanoflagellates, also show response behaviors to light input [2]. Because of their small body size and lack of a nervous system, the light-response network of these organisms is established mainly within the intracellular space. On the other hand, along with the increase in body size and the acquisition of a nervous system during animal evolution, it is expected that organisms started to establish and behave with an intercellular network involving photoreceptors, nervous system components and motor organs/organelles. For example, diel vertical migration (DVM) is a well-known behavior through which zooplankton stay at the surface at night and sink deeply into the ocean during the daytime. This behavior has been explained as a means by which zooplankton escape from diurnal predators such as fish and harmful ultraviolet (UV) light during the daytime [3, 4], and it has been suggested that the behavior is driven by an intercellular network. One of the most studied model organisms for DVM so far is *Daphnia*, and analyses of the migrations of *Daphnia* away from predators and UV light have been well reported. For example, stronger UV light induces deeper migration of *Daphnia* [3–5]. As photoreceptors for these metazoan behaviors, opsin family members function mainly to receive light of a specific wavelength and transmit the signal to downstream systems [6, 7]. Opsins, which are found only in the metazoan group [8], belong to the group of sensory G-protein-coupled receptors (GPCRs) and are categorized into visual/nonvisual types [7]. The motor organs/organelles, as the effectors of light responses, are muscle in most macroscopic animals and cilia in relatively small aquatic animals. Light-induced muscle activities, such as those involved in the pupillary light reflex of our eyes, the light avoidance behavior of earthworms, and the phototaxis of insects, have been well investigated [9–11]. Muscle-based light responses have been reported throughout the bilaterians [6, 9–15]. On the other hand, in the planktonic larvae of flatworms, Annelida, and Mollusca, responses to light are supported mainly by changes in ciliary beating, with which the larvae show reflex behavior and/or phototaxis [16–18]. This cilia-based response to light is also found in cnidarian and sponge larvae [19, 20], although it is expected that the sponge lineage lost Opsin genes [8]. Even though ctenophores and placozoa have motile cilia, they do not show the cilia-based response to light [21, 22]. Although nematodes and arthropods have lost motile cilia in their clades and of course have no cilia-based responses to light, it is obvious that the light-induced response of cilia is a shared characteristic within protostomes and cnidarians. However, it is unclear whether this pathway is conserved among neuralians because cilia-based responses to light are poorly reported and understood in deuterostomes, resulting in a lack of understanding of the signaling pathway from light to cilia in these groups. This lack of clarity might be because the body size of deuterostomes is relatively large, which makes the behaviors of these animals dependent on muscle activities and masks subtle ciliary activity if it is present.

Among deuterostomes, some echinoderms and hemichordates have free-living planktonic embryos/larvae, which are the only groups that move with mainly cilia rather than muscle. Therefore, they are candidates from which we might obtain information on the presence and mechanisms of cilia-based responses to light input in deuterostomes. In particular, sea urchin larvae are ideal experimental model organisms for this purpose because they do not use muscle at all for their swimming behavior and are suitable for genetic analysis. The sea urchin genomes contain a maximum of 9 opsin genes (e.g., Opsin1, Opsin2, Opsin3.1, Opsin3.2, Opsin4, Opsin5, Opsin6, Opsin7, and Opsin8 in *Strongylocentrotus purpuratus*), which belong to a group of sensory GPCRs, and most of them are categorized into reported opsin groups, except for Opsin2 and Opsin5 [23]. Opsin2 and Opsin5 have been found only in echinoderms; therefore, they are thought to be specific to the group. Because some of the expression patterns of these genes have been reported in both embryos/larvae and adults [24–27], it is reasonably expected that sea urchins have the ability to react to light stimuli from a genomic perspective, as reported in adult behavioral studies [28, 29]. However, the functions of photoreceptor genes have never been confirmed by using genetic modification except for the function of the recently reported gut-regulatory Go-Opsin [12], and the neural pathway regulating cilia-based larval behavior has not yet been identified. Therefore, in this study, we sought to describe how the swimming behavior of sea urchin larvae responds to light and to identify the neural pathway involved in the response.

## Results

**Fig 1:**
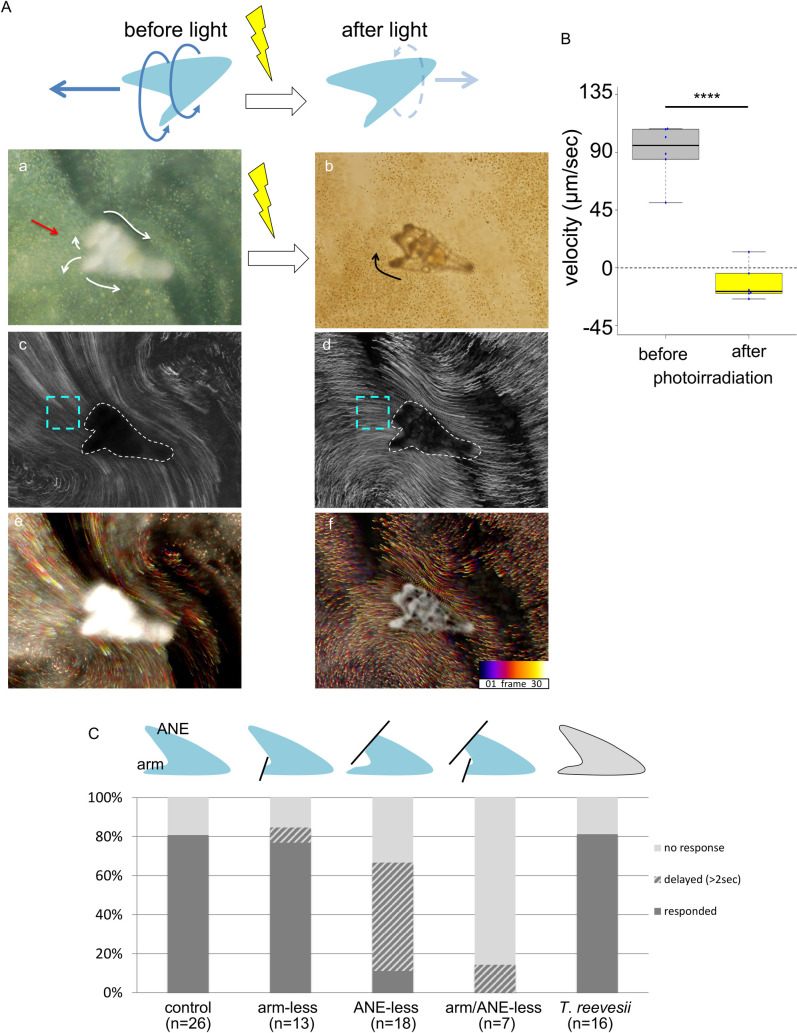
Sea urchin larvae stop swimming forward and swim backward in response to a light stimulus. (A) Schematic images showing a change in swimming behavior after photoirradiation. The images captured from S3 Video show the changes in the movement of diatom particles around larvae after photoirradiation (*cf*. Aa to Ab). Ac and Ad show the superimposed images for two seconds each before (Ac) and after (Ad) photoirradiation. The sky-blue rectangle indicates the region of interest (roi) we used for the calculation of particle velocity. Ae and Af show the temporal-color-code mode for the superimposed images. As shown in the indicator, dark blue and bright yellow mark the beginning and end of the 2 sec movie before (Ae) and after (Af) photoirradiation, respectively. (B) Particle velocity measured before and after photoirradiation. N = 6 in each experiment. The velocities of 10 particles were scored for each N. ****, *p*≤0.0001. (C) Larval arms are required for photoreception. The graph shows the percentages of the larval responses to photoirradiation. The images on each bar graph indicate the micromanipulated larvae used for each experiment. ANE, anterior neuroectoderm.

To investigate whether sea urchin larvae respond to light, we irradiated the larvae with strong light in a dish in which they had previously stayed at the surface of the seawater. We then observed their behaviors. Intriguingly, the larvae dropped from the surface immediately upon photoirradiation, and some of them swam backward (S1 Fig, S1 Video). Larvae of different species, such as *Hemicentrotus pulcherrimus* and *Temnopleurus reevesii*, showed similar behaviors (S1 Fig, S2 Video), suggesting that this response is common in some sea urchin groups. To visualize and quantify this larval behavior, we measured the velocities of diatom particles in front of the larvae that were attached and immobile on glass before and after photoirradiation (Fig 1A, see Materials and Methods). The diatom movements reflected the water currents that were produced by the ciliary beating of the larvae [30]. Although the larvae swim in a three-dimensional world, we measured their behaviors under a microscope in a two-dimensional plane in this study to obtain data that can be reproduced with conventional equipment in ordinary laboratories, but we will try in future analyses to detect swimming behavior with a high-speed tracking 3D microscope. In the absence of strong light, the larvae generally swam forward (Fig 1B), but after strong photoirradiation, a weakly reversed water current was produced, indicating that the larvae had stopped swimming and/or swum backward in response to light (Fig 1B, S3 Video). The average velocities of the water current toward the larvae, which reflected larval forward swimming, were 90.63 μm/sec (before light, N = 6, n = 10 each) and -12.19 μm/sec (after light, N = 6, n = 10 each).

Because it is reasonable to speculate that the cessation of forward swimming and/or the reversal of swimming direction after photoirradiation is mediated by a complex system including photoreceptors, neurons, and ciliary cells, we attempted to identify the most important body parts for this response by removing the larval anterior neuroectoderm (ANE) and/or postoral arms and then observing larval swimming behaviors. It has already been shown that this microdissection method is useful for investigating the essential parts of the body for a targeted phenomenon [12]. The cessation of forward swimming or reversal of swimming direction after photoirradiation was observed in 80% of the normal *H*. *pulcherrimus* larvae and in the same percentage of *T*. *reevesii* larvae (Fig 1C). This quantitatively supports the idea that this phenomenon is common among some sea urchin groups. When we removed the postoral arms, the manipulated larvae showed a response to photoirradiation similar to that of the control larvae (Fig 1C). This result suggested that the postoral arms, which contain ciliary bands and some neurons, do not seem to be important for the response to photoirradiation. Larvae from which the ANE and preoral arms were removed stopped swimming forward and/or swam backward upon photoirradiation, but the timing was delayed (>2 sec) (Fig 1C). This result indicates that the ANE is more important for the response to photoirradiation than the postoral arms. However, compared with that of control larvae, the response of ANE-removed larvae was weak, suggesting that the ANE is not the only tissue essential for the response to light. Intriguingly, when both the ANE and postoral arms were removed, the larvae swam only forward and did not strongly respond to photoirradiation (Fig 1C). These data indicate that some tissues, such as the nervous system near the ANE and/or postoral arms, play an important role in the photoirradiation response.

**Fig 2:**
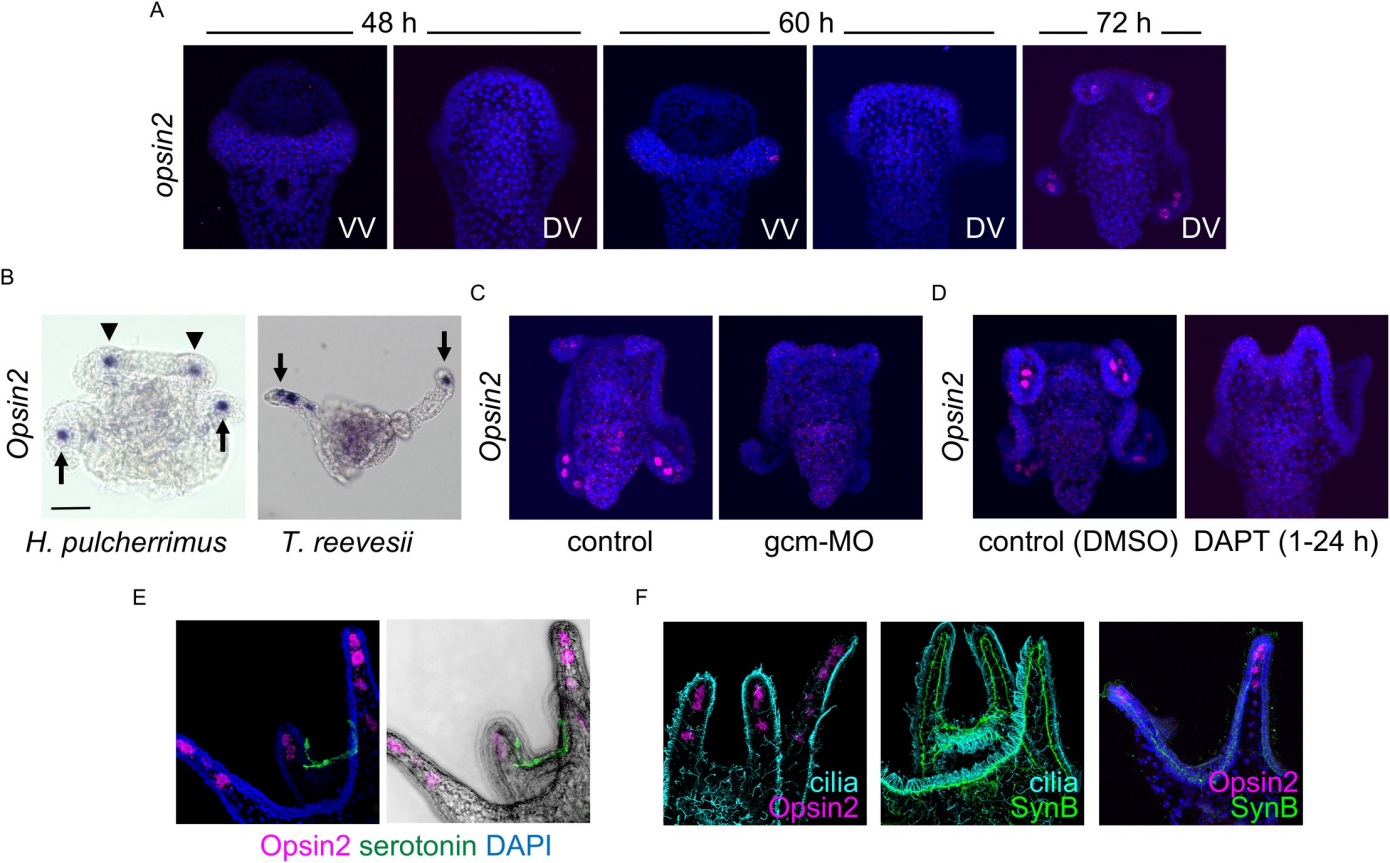
Opsin2 expression during embryogenesis. (A) Fluorescent *in situ* hybridization of *Opsin2* mRNA. Although 48-hour-old larvae showed no mRNA signal, 72-hour-old larvae clearly had *Opsin2* expression at the tips of their arms. VV, ventral view; DV, dorsal view. (B) Chromogenic *in situ* hybridization of *Opsin2* mRNA in pluteus larvae of *H*. *pulcherrimus* and *T*. *reevesii*. Bar = 20 μm. The arrows and arrowheads show *Opsin2*-expressing cells in the postoral arms and preoral arms, respectively. The *Opsin2*-expressing cells in the preoral arms of *T*. *reevesii* are out of focus in this image. (C, D) The expression of *Opsin2* was abrogated in larvae in which the secondary mesenchymal cell specifier Gcm (C) or Delta-Notch signal (D) was attenuated. (E) Opsin2 protein was expressed in the same cells expressing *Opsin2* mRNA. Green shows serotonergic neurons. Blue in the left image shows the nuclei. The fluorescent image is merged with the brightfield image on the right. (F) Opsin2-expressing cells were close to the ciliary band and neurons. Major neuronal axons, which express the panneural marker synaptotagmin B (SynB), were located beneath the ciliary band.

**Fig 3:**
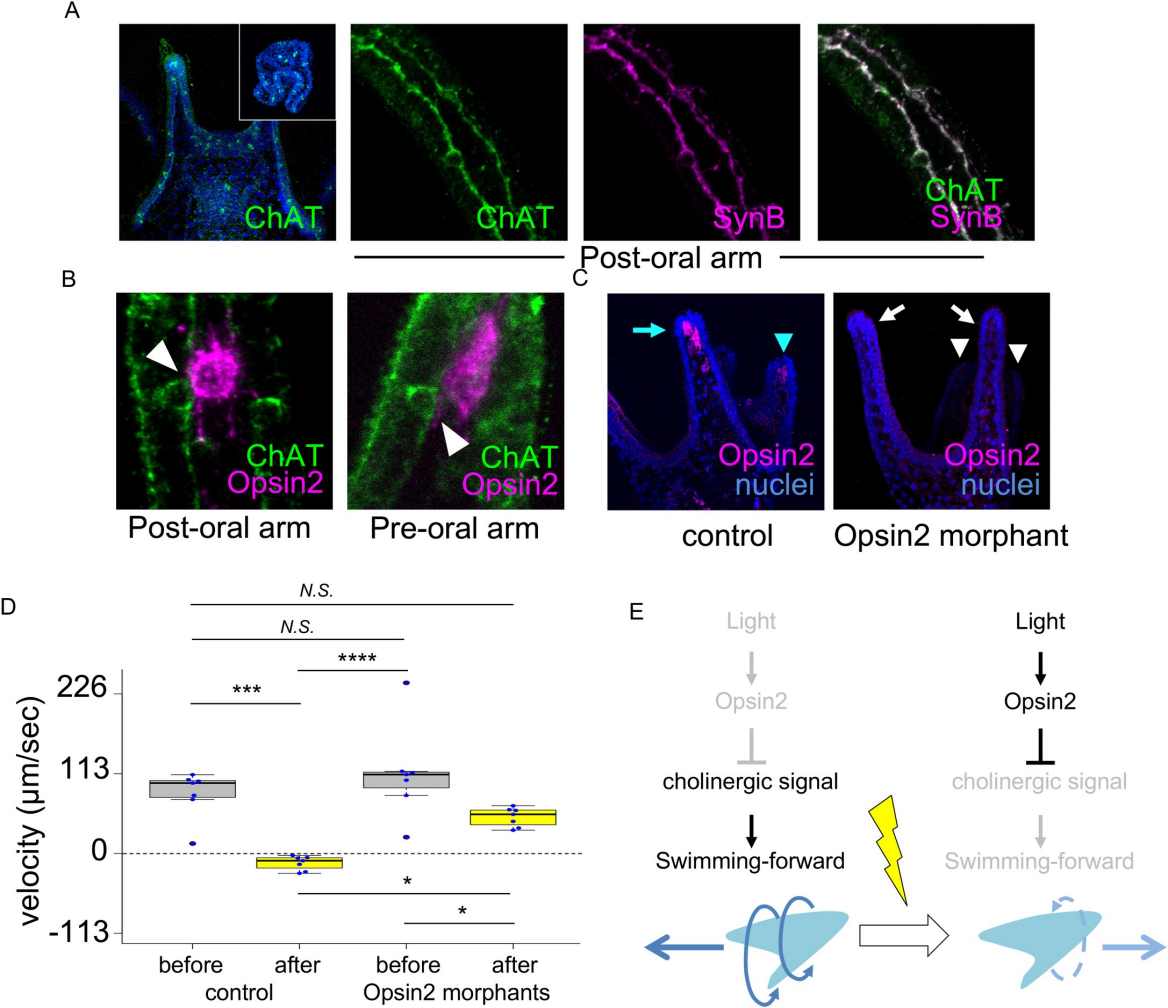
Opsin2, which is expressed in mesenchymal cells located in the arms, is required for photoreception. (A) Immunohistochemical analysis of the ChAT protein. The ciliary band neurons in the postoral arms are cholinergic. (inset) *In situ* hybridization of *choline acetyltransferase* (*ChAT*) mRNA in pluteus larvae. (D) The Opsin2 protein was not translated in Opsin2-MO2 morphants. (B) Details of the relationship between Opsin2-expressing cells and cholinergic neurons. The cell processes from Opsin2-expressing cells appear to attach or be closely adjacent to neurons (arrowhead). (C) The Opsin2 protein was expressed in mesenchymal cells in the postoral arms (sky-blue arrow) and preoral arms (sky-blue arrowheads), and it disappeared after morpholino injection (white arrows and arrowheads). (D) The graph shows the difference in particle velocity before vs. after photoirradiation in the presence or absence of the Opsin2 protein. The particle velocity was measured before and after photoirradiation. N = 7 in each experiment. The velocities of 10 particles were measured for each N. *, *p*≤0.05, **, *p*≤0.01, ***, *p*≤0.001, ****, *p*≤0.0001, *N*.*S*., not significant. (E) Schematic images summarizing the results of this study. Under dark or weak-light conditions (left), the cholinergic system drives forward swimming, but strong photoirradiation (right) stops the cholinergic system via Opsin2.

Among the members of the complex system that is expected to be critical for this light-dependent phenomenon, neurons and cilia are present mainly in the ciliary band and throughout the entire body [31, 32], respectively, but the spatial expression patterns of photoreceptors in *H*. *pulcherrimus* had not been reported until recently. Since the temporal and spatial patterns of the larval Opsins *Opsin2* and *Go-Opsin* have been reported in *Strongylocentrotus purpuratus* [25, 33], we tried to detect the expression patterns of these molecules in *H*. *pulcherrimus*. Similar to the pattern in *S*. *purpuratus*, we found that the *Go-Opsin* gene was expressed exclusively around the ANE; it was not expressed in the arms of *H*. *pulcherrimus* [25, 33], indicating that it is not a strong candidate as the factor that focuses swimming behavior. In addition, we have already reported that Go-Opsin is involved in the light-dependent gut regulatory system [12]. On the other hand, *Opsin2* was expressed in mesenchymal cells at the tips of the postoral arms (Fig 2A and 2B, arrows) and the preoral arms adjacent to the ANE (Fig 2A and 2B, arrowheads). Identical expression patterns were observed in *T*. *reevesii* (Fig 2B, arrows, although the preoral arms are out of focus in this image). Because the mesenchymal cells at the tips of the arms include both primary mesenchymal cells (PMCs) and secondary mesenchymal cells (SMCs), to confirm which lineage expresses *Opsin2*, we blocked the specification of SMCs by attenuating the function of the specifier gene, glial cells missing (gcm) [34], with a specific morpholino. *Opsin2* mRNA was not detected in gcm morphants (Fig 2C). In addition, in larvae treated with a gamma-secretase inhibitor (DAPT: *N-*[*N-*(3,5-difluorophenacetyl-L-alanyl)]-(*S*)-phenylglycine *t*-butyl ester), in which the SMC specification signal transmitted via Delta/Notch signaling was blocked during early embryogenesis, *Opsin2*-positive cells were not present (Fig 2D). These data strongly indicate that *Opsin2*-positive mesenchymal cells belong to the SMC lineage. These patterns are different from those in *S*. *purpuratus* [33], possibly because of species differences. To confirm our *in situ* hybridization data showing targeted *Opsin2* mRNA expression, we generated a specific antibody against Opsin2 protein produced in *Escherichia coli* (see Materials and Methods). The specificity of the antibody is shown in Figs 3C and S2. Immunohistochemistry using this antibody showed that the Opsin2 protein was present in the same mesenchymal cells that expressed *Opsin2* mRNA (Fig 2E). This finding strongly indicates that Opsin2 mRNA and protein are expressed in the SMC lineage at the tips of both the postoral and preoral arms in sea urchin larvae.

It has been suggested that swimming behaviors of sea urchin larvae are regulated mainly by a nervous system underlying the ciliary band (Fig 2F) [30, 32], and Opsin2-expressing cells are located close to the ciliary band. However, since no papers have reported experimental results indicating the involvement of ciliary band neurons in swimming behaviors, we next investigated whether ciliary band neurons regulate larval movements under normal conditions. As shown in Fig 2F, Synaptotagmin B (SynB)-positive neurons were distributed under the entire ciliary band. A recent paper reported that some ciliary band neurons are cholinergic in larvae of the sea urchin *Lytechinus variegatus* [35]. To confirm whether the same type of neuron was present under the ciliary band in our model sea urchin, *H*. *pulcherrimus*, we performed *in situ* hybridization using an RNA probe specific for *choline acetyltransferase* (*ChAT*) mRNA. The results showed that *ChAT*-expressing cells were likely located under the ciliary band, as reported in *L*. *variegatus* (Fig 3A, inset). However, because of the shrunken shapes of the pluteus larvae, it was difficult to identify the detailed expression patterns. Therefore, we developed a specific antibody against the ChAT protein (see Materials and Methods) and observed the expression patterns. The specificity of the antibody is shown in S3 Fig. The results of this observation showed that most ciliary band neurons seemed to be cholinergic, especially in larval arms (Fig 3A). These results suggest that ciliary beating controlling larval swimming behaviors involves the activity of cholinergic neurons. To confirm this supposition, we tried to knock down the activity of ChAT using a specific morpholino and succeeded in blocking translation, as shown in S3A Fig. However, this blockade had multiple effects on larval behaviors (which will be described in a later publication), leading us to conclude that simply knocking down the cholinergic neural pathway is not suitable for analyzing swimming behavior. Thus, we applied inhibitors of acetylcholine receptors and assessed swimming behavior. In sea urchin genomes, two types of acetylcholine receptors have been annotated: muscarinic acetylcholine receptor (mAChR) and nicotinic acetylcholine receptor (nAChR) [36]. When we blocked mAChR using the specific inhibitor atropine, the larvae did not show a normal response to light due to their slight forward swimming (S4A Fig); some of them exhibited only backward swimming. To confirm the findings, we applied a lower concentration of atropine and traced and measured the swimming behavior. Compared with the distance of movement over 45 sec of the control larvae, that of the atropine-treated larvae was significantly shorter (S4B and S4C Fig). In addition, another inhibitor of mAChR, scopolamine, had a similar effect on larvae as atropine treatment (S4B and S4C Fig). In contrast, when we blocked nAChR using the specific inhibitor d-tubocurarine, the larvae showed a normal response to light (S4A Fig). These results suggest that cholinergic neurons and mAChR are required for normal forward swimming. Based on the temporal expression patterns of a member of annotated *mAChRs* in *S*. *purpuratus*, *mAChR-M5* (SPU_016177) is expressed during embryonic and larval stages [37]. *In situ* hybridization using an antisense probe for HPU_21265, a homolog of SPU_016177 in *H*. *pulcherrimus* [27], revealed that *mAChR* was expressed in the anterior half and mainly at the oral ectoderm, which was surrounded by the ciliary band (S3D Fig). Because of the presence of cilia on all ectodermal cells, it has been suggested that larval swimming patterns and directions are likely regulated by complex combinations of ciliary beating in different areas [30, 38]. These patterns of forward swimming and the movement of the water in front of the larvae, on which we focused in this study, reflect a process involving cholinergic neurons and mAChR.

Detailed observations revealed that ChAT-positive ciliary band neurons appeared to be directly associated with or closely adjacent to Opsin2-expressing cells in both the preoral and postoral arms (Fig 3B, arrowheads). To examine whether Opsin2 plays an essential role as a photoreceptor in the observed phenomenon, we knocked it down with specific morpholino oligonucleotides (Opsin2-MO) (Fig 3C [white arrows and arrowheads], S2) and observed larval swimming behavior. The control larvae normally stopped swimming forward or swam backward in response to photoirradiation, whereas the Opsin2 morphants showed only weakened forward swimming (Fig 3D, S4 Video). The average velocities of the water current toward the larvae were 84.29 μm/sec (before light in the control group, N = 7, n = 10 each), -13.49 μm/sec (after light in the control group, N = 7, n = 10 each), 113.12 μm/sec (before light in the Opsin2 morphant group, N = 7, n = 10 each), and 51.43 μm/sec (after light in the Opsin2 morphant group, N = 7, n = 10 each). This result strongly suggests that Opsin2 is the photoreceptor that mediates light-dependent arrest/reversal of swimming, possibly by inhibiting the activity of cholinergic neurons. In addition, because all Opsin2-positive mesenchymal cells are located at the tips of the postoral and preoral arms, the finding that Opsin2 morphants continued swimming forward supports the finding that the armless larvae kept swimming forward under photoirradiation conditions (Fig 1C). On the other hand, the fact that weakened forward swimming was observed in Opsin2 morphants suggests that some other components, such as other opsins and/or nervous system pathways, might be involved in the change in swimming behavior upon photoirradiation. The detailed mechanisms by which these cells communicate with each other, including by direct or indirect contact, will be studied in the future.

## Discussion

Based on the data shown here, under normal conditions, sea urchin larvae use cholinergic neurons to drive forward swimming; however, when the larvae face strong photoirradiation, the activated photoreceptor Opsin-2 seems to suppress the activity of cholinergic neurons, causing forward swimming to be stopped/weakened and promoting backward swimming (Fig 3E). Although the details of this signal transduction pathway from Opsin-2 cells to cholinergic neurons have not yet been revealed, our data clearly show that sea urchin larvae respond to light stimuli, as reflected by their swimming behavior. The presence of this pathway from light to stopping/reversal of swimming through secondary mesenchymal photoreceptors and the ciliary band nervous system was strongly supported by multiple knockdown experiments using MOs and pharmacological experiments. In addition, this phenomenon is likely common, at least in some sea urchin species, since both *H*. *pulcherrimus* and *T*. *reevesii* showed the same response to photoirradiation and the same localization of Opsin2 cells (Figs 1C and 2B). Because all echinoderm larvae mainly use cilia, not muscles, in their swimming behavior and because the ciliary ectoderm of these larvae is associated with the nervous system [32, 39, 40], it is likely that all echinoderm groups have a similar light-induced ciliary response. In fact, the larvae of brittle stars show DVM behavior, in which they swim down to the deep ocean during the daytime and swim up to the surface during the night [41], suggesting that their ciliary beating is controlled in response to light. Since it has rarely been proven that a cilia-based response to light input is present in other deuterostomes by experiments thus far [42, 43], it is of interest that sea urchins have the ability to respond to light with cilia via an opsin-nervous system network. Since the appearance of opsin genes at the metazoan stem [8], it is speculated that most photoreception systems have become dependent on opsin proteins in metazoans. In addition, it is also expected that the appearance of neurons helped enable long-distance intercellular communication between photoreceptors and motor organs/organelles, especially in neuralians. Because cnidarians and ciliated protostome larvae utilize mainly the light-induced response of cilia in the regulation of their behaviors [8, 16, 17], the light-induced response of cilia was likely already present prior to the appearance of the mesodermal muscles that play essential roles in bilaterian movements. Without experimental or detailed observation data in chordates, it is still unclear whether the light-induced response of cilia is commonly present or absent in the deuterostome lineage, although the pathway has now been found in the sea urchin group. One scenario is that the light-induced response of cilia is conserved among deuterostomes but still unidentified. In hemichordates, which belong to Ambulacraria together with the echinoderms, it has been reported that adults respond to light and move to avoid it with muscle [13], but larval behaviors away from light have rarely been an experimental research focus. Indirectly developing larvae swim with neuron-associated cilia like echinoderm larvae [44], and some of them have clearly pigmented eyespots [45]. In addition to the presence of opsin genes in their genomes [23, 46] and the similarity of their developmental steps to those of echinoderms, these morphological characteristics suggest that the larvae of hemichordates might have cilia-based responses to light stimuli. On the other hand, because muscular movement is so conspicuous in the chordate lineage, it is possible that the subtle responses of tiny cilia are masked and/or have never been focused on even if cilia-based responses to light are present. Although it is still unclear whether the light-cilia relationship observed in sea urchin larvae is conserved in vertebrates, in human behavior, there is a phenomenon that might be comparable: the photic sneeze reflex. This is a phenomenon in which humans have a sneezing reflex when they are exposed to strong photoirradiation, such as sunlight. Although the detailed molecular mechanisms responsible for the photic sneeze reflex have not yet been investigated [47], it is possible that dysfunction of ciliary beating is induced by photoirradiation, as in sea urchin systems, and causes the sneezing reflex in the absence of any debris or irritants. In particular, ciliary beating in the nose/nasal cavity is controlled by cholinergic neurons, and it has been suggested that dysfunction of these neurons is the reason for the photic sneeze reflex [48]. It is of interest if the cessation of cholinergic neuron signaling and the consequent sneeze reflex are induced by photoirradiation, since our data might provide hints to help determine whether the light-induced response of cilia is present in vertebrates and whether the involved system is conserved between echinoderms and vertebrates. In addition, cilia are present inside of the ventricular system in human brain, and cerebrospinal fluid flows via cilia to transport neurotransmitters/neuromodulators, meaning that cilia are one of the key factors for the functions of the brain [49]. If similar responses of cilia to light occur in the ventricular system, the signaling pathway found in sea urchin larvae might help elucidate the mechanisms of human behaviors and/or feelings in response to light.

To date, some characteristics of the swimming behaviors of sea urchin larvae have been reported. For example, the larvae swim against gravity, and this negative gravitaxis is managed by serotonergic neurons, which are exclusively located in the ANE region [30]. However, because of the lack of knowledge on how sea urchin larvae detect gravity, the signaling pathway that is initiated by gravity and is transmitted through serotonergic neurons remains unknown. In addition, previous works have analyzed larval behaviors by considering larvae as particles moving against gravity [50]; however, it is difficult to describe the detailed signaling pathway in the nervous system based on detection of gravity in combination with ciliary beating. Similarly, although sea urchins respond to external and environmental stimuli, the detectors and critical nervous system components have not been well described for a long time, except for the Go-Opsin>serotonergic neurons>nitric oxide pathway, which was recently described in a publication [12]. Another study has reported that dopamine can induce backward swimming in sea urchin larvae [51]. This neurotransmitter may be a candidate controller of swimming cessation and/or backward swimming under photoirradiation; however, larvae lacking the postoral arms, in which dopaminergic neurons are present [52], had a normal response (Fig 1), suggesting that dopamine is not key to this behavior. Although the details regarding how light information is transmitted to and stops the activity of cholinergic neurons are still unclear, the data presented in this paper show that light-exposed Opsin2-expressing cells inhibit constitutively activated cholinergic signaling. Opsin2-expressing cells appear to directly communicate with cholinergic neurons, but we cannot completely exclude the possibility that Opsin2-expressing cells secrete unknown factors that inhibit mAChR. Because the beating of respiratory cilia in mammals and palate/esophageal cilia in frogs is driven by an activated cholinergic system through mAChR and because antagonists of mAChR inhibit ciliary beating [53–56], some parts of the relationship between the mAChR-based cholinergic system and cilia seem to be conserved in vertebrates and sea urchins.

## Materials and methods

### Animals (sea urchins)

Adults of *Hemicentrotus pulcherrimus* were collected around the Shimoda Marine Research Center of the University of Tsukuba and around the Marine and Coastal Research Center of Ochanomizu University. Adults of *Temnopleurus reevesii were collected around the Shimoda Marine Research Center of the University of Tsukuba*. *Both species were collected under the special harvest permission of the prefectures and Japan fishery cooperatives*. *They were kept in temperature-controlled aquariums (13°C and 24°C for H*. *pulcherrimus and T*. *reevesii*, *respectively) until use*, *and* the used adults were kept until the next breeding season or released to the place from which they were collected. *Gametes were collected by* intrablastocoelic *injection of 0*.*5 M KCl*, *and the embryos/larvae of H*. *pulcherrimus and T*. *reevesii were cultured at 15*°C and 22°C, respectively, in glass beakers or plastic dishes that contained filtered natural seawater (FSW) with 50 μg/ml kanamycin. In some experiments, we fed 10 μl of SunCulture algae (*Chaetoceros calcitrans*, Marinetech, Aichi, Japan, approx. 30,000 cells/μl) to the larvae as forage in 3.0 ml of FSW almost every day.

### Photoirradiation procedure and observation of larvae

In each experiment, pluteus larvae at appropriate stages (4–5 days post-fertilization) were attached to poly-L-lysine (Merck)-coated glass slides with a drop (5.0 μl) of seawater. To visualize the water current, 0.5 μl of SunCulture algae (*Chaetoceros calcitrans*) was added to the seawater drop. The specimen was moved onto an IX-70 microscope (Olympus, Tokyo, Japan) equipped with a DP-71 digital camera (Olympus) under ambient room light (7.5–13.0 μmol m^-2^ s^-1^), which does not induce cessation of forward swimming. Photoirradiation of the specimens was performed by turning on the light of a microscope without any neutral density filters. The photon flux density was adjusted to 1425 μmol m^-2^ s^-1^.

### Microscopy and image analysis, including calculation of the velocity of diatoms in front of the larvae

Specimens were observed using a fluorescence microscope (IX70, Olympus, Tokyo, Japan), a confocal laser scanning microscope (FV10i, Olympus), and a dissecting microscope (M165C, Leica Microsystems GmbH, Wetzlar, Germany). Superimposed images were made with Fiji (Z-projection with the standard deviation option), and the time-encoding with color to show the direction of the particles was accomplished with the temporal-color-code program from Kota Miura (https://github.com/fiji/fiji/blob/master/plugins/Scripts/Image/Hyperstacks/Temporal-Color_Code.ijm). For Figs 1B and 3D, videos of the specimen were captured 5 sec before and 10 sec after photoirradiation. The videos were opened with Fiji (https://imagej.net/Fiji) and analyzed with the Particle Track and Analysis (PTA) plugin (https://github.com/arayoshipta/projectPTAj). Initially, we chose an roi at the front of the larva (dotted-line rectangle, Fig 1Ac and 1Ad) and cropped it. Then, the PTA plugin was run for the roi. The velocity of the diatoms was calculated by dividing the travel distance (μm) by the time (sec). The velocity was calculated with the direction toward the larva as the positive direction and the direction away from the larva as the negative direction. The velocities of the 10 diatoms with the longest migration in each sample were averaged to obtain the average velocity of each larva. To compare experimental specimens with controls, we used Welch’s *t* test (two-tailed) or one-way ANOVA followed by Tukey’s post hoc test with a significance level of 0.01 or 0.05. For Figs 1C and S4A, we counted the number of larvae that responded (stopped swimming forward or swimming backward) or did not respond to photoirradiation (continued swimming forward), and the ratio was calculated. These samples were dissected by micromanipulation or drug-treated. The swimming distance was also measured for S4B and S4C Fig; Fiji was used for the superimposed images. The panels and drawings for the figures were made using Adobe Photoshop and Microsoft PowerPoint.

### Reagent treatments

3,5-Difluorophenylacetyl-L-alanyl-L-S-phenylglycine T-butyl ester (DAPT; Sigma–Aldrich, St Louis, MO, USA), which was used as a γ-secretase inhibitor, was prepared as a 20 mM stock in dimethyl sulfoxide (DMSO) and diluted in FSW to 20 μM before use [57]. To obtain larvae in which the secondary mesenchymal cells failed to develop completely, we treated the larvae with DAPT from 1 hour to 24 hours after fertilization [58]. The same volume of DMSO was applied as a control. Atropine sulfate and d-tubocurarine chloride were used as mAChR and nAChR inhibitors, respectively. The atropine and d-tubocurarine were dissolved in distilled water (DW) as 100 mM stocks and diluted into the larval culture medium 1 min before observation.

### Whole-mount *in situ* hybridization and immunohistochemistry

Whole-mount *in situ* hybridization was performed as described previously [59] with some modifications. A cDNA mix from several embryonic stages was used to isolate the targeting genes to make RNA probes based on the *H*. *pulcherrimus* genome and transcriptome [27]. Digoxygenin (Dig)-labeled RNA probes were generated from PCR amplicons with SP6 and T7 primers from pCS2+ChAT and pCS+mAChR. The samples were fixed with 3.7% formalin (Merck) containing FSW overnight at 4°C. They were washed with MOPS buffer (0.1 M MOPS pH 7.0, 0.5 M NaCl, 0.1% Tween-20) seven times for 7 min each at room temperature (RT) and rinsed with hybridization buffer (70% formamide, 0.1 M MOPS pH 7.0, 0.5 M NaCl, 1 mg/ml BSA, 0.1% Tween-20) three times. Then, the samples were incubated in hybridization buffer with Dig-labeled RNA probes (0.4 ng/μl final concentration) of *ChAT* (HPU_01496) and *mAChR* (HPU_21265) at 50°C for 5–7 days. After washing with MOPS buffer seven times at RT, three times at 50°C, and twice at RT, the Dig-labeled probes were detected with an anti-Dig POD-conjugated antibody (Roche) for *ChAT* overnight at 4°C and treated with a Tyramide Signal Amplification Plus System (TSA; PerkinElmer, Waltham, MA, USA) for 8 minutes at RT. When observed, the samples were incubated in MOPS buffer containing 2.5% 1,4-diazabicyclo-2-2-2-octane (Sigma–Aldrich) to prevent photobleaching. For *mAChR*, after washing, the Dig-labeled probes were detected with an anti-Dig AP-conjugated antibody (Roche) for 1 hour and washed with MOPS buffer overnight. The samples were washed three times in alkaline phosphatase buffer (0.1 M Tris-HCl pH 9.5, 0.1 M NaCl, 50 mM MgCl_2_, 1.0 mM levamisole, and 0.1% Tween-20), and the signal was detected with 5-bromo-4-chloro-3-indolyl-phosphate/nitro blue tetrazolium (BCIP/NBT) in AP buffer containing 10% dimethylformamide.

Whole-mount immunohistochemistry was also performed as described previously [59] with some modifications. Cold methanol-fixed (10 min) samples were blocked with 1% skim milk in PBST for 1 hour at RT and incubated with primary antibodies (dilutions: mouse anti-Synaptotagmin B (SynB) [32] 1:100, rabbit anti-acetylated-α-tubulin 1:800 (Cell Signaling Technology), mouse anti-Opsin2 1:200 (see below), and rabbit anti-Opsin2 1:100 (see below)) overnight at 4°C. The primary antibodies were detected with Alexa 488- or Alexa 555-conjugated secondary antibodies (Thermo Fisher Scientific, Waltham, MA, USA).

### ChAT and Opsin2 antibodies

cDNA encoding the first 86 amino acids of ChAT and amino acids 784–1395 of Opsin2 were cloned into the pET32a vector (Novagen, Darmstadt, Germany), and the histidine-tagged proteins were produced in ArcticExpress (DE3) bacteria with a final concentration of 1.0 mM isopropyl ß-D-thiogalactopyranoside (IPTG) for 4 hr at RT. The bacteria were lysed with 8 M urea, and the fusion proteins were purified by Ni-column chromatography (Cytiva, Tokyo, Japan) and used to immunize three mice under the permission of the Animal Care Committee of the University of Tsukuba (No. 19–65, 1040). Using the same purified ChAT protein, rabbit antisera were produced by Eurofins Genomics (Tokyo, Japan). The antisera were screened by whole-mount immunohistochemistry, and IgG was purified with Melon gel (Thermo Fisher Scientific) and used for experiments.

### Microinjection of morpholino antisense oligonucleotides (MOs)

For microinjection, we used injection buffer (24% glycerol, 20 mM HEPES pH 8.0 and 120 mM KCl). The morpholino (Gene Tools, Philomath, OR, USA) sequences are in the reagent table, and the in-needle concentration with injection buffer was 1.0 mM. Two nonoverlapping translation-blocking morpholinos for Opsin2 and ChAT were used to confirm the function specificity (S1 and S2 Figs). For negative control experiments, we injected a random MO or only injection buffer. The concentrations of MOs in the needles were as follows:

Opsin2-MO1 (0.4 mM), 5’-AGTTTGCCATCTTTGTGTTGCTTCG -3’

Opsin2-MO2 (0.4 mM), 5’-CGCCAATAACCACTGATCACAGTCG -3’

ChAT-MO1 (0.2 mM), 5’-ACGATTAGGCATGTGGTTCATGTAT -3’

ChAT-MO2 (1.0 mM), 5’-TGGAACGTCCAATAGTGGTATTGTA -3’

Gcm-MO, 5’-GCTTTGGACTAACCTTTTGCACCAT -3’ [34], and

Random-MO (1.0 mM).

Microinjections into fertilized eggs were performed as previously described [60, 61]. After microinjection, the embryos were washed with FSW three times and stored with 50 *μ*g/ml kanamycin until the desired stages were reached.

### Micromanipulation

Larvae were placed in 6 cm plastic dishes filled with seawater, and the postoral arms or ANE/preoral arms were cut off with an injection needle under a dissecting microscope (Leica M165). The dissected larvae were transferred to new seawater and used for experiments.

## Supporting information

S1 Fig Larvae of both *Hemicentrotus pulcherrimus* and *Temnopleurus reevesii* move away from the water surface when illuminated. These are captured images from S1 and S2 Videos. The times (sec) shown on the images indicate the timing before and after photoirradiation.
(PDF)

S2 Fig Confirmation of morpholino specificity. The Opsin2 protein was not detected in Opsin2-MO2 morphants.
(PDF)

S3 Fig Specificity of the ChAT antibody. (A) Two nonoverlapping morpholinos blocked the translation of ChAT. (B) Using the same antigen, we made an anti-ChAT antibody in a rabbit. Although the background was slightly high, the antibody recognized ciliary band neurons to a similar degree as a mouse antibody. The middle image is a magnified region of the dotted-line rectangle on the left. The right image shows the disappearance of the rabbit anti-ChAT antibody signal in the ChAT-MO1 morphant, supporting the specificity of the antibody.
(PDF)

S4 Fig Normal forward swimming of sea urchin larvae depends on cholinergic neurons. (A) The graph shows the change in particle direction before and after photoirradiation in the presence of acetylcholine and antagonists of its receptors. Excess acetylcholine inhibited the larval response to light. Because atropine-treated larvae basically did not show forward swimming, they did not respond to light. The nicotinic acetylcholine inhibitor d-tubocurarine did not inhibit the larval response to light input. (B) Superimposed images of 45 sec of swimming behaviors of sea urchin larvae (control [water-], atropine-, and scopolamine-treated). (C) The graph shows the distance of larval swimming shown in (C). Water-treated larvae (n = 20) could swim significantly longer distances than atropine-treated (n = 20) or scopolamine-treated (n = 14) larvae. *, *p*≤0.05, ****, *p*≤0.0001, *N*.*S*., not significant. (D) *In situ* hybridization for *mAChR*. mRNA of *mAChR* was expressed in the anterior regions of the larvae. The ciliary band region and posterior end (asterisks) had no strong expression.
(PDF)

S1 Video Response to photoirradiation in larvae of *Hemicentrotus pulcherrimus*. The larvae stay at the surface of the seawater for a long time under dark conditions, but at the moment of photoirradiation, they leave that position.
(MP4)

S2 Video Response to photoirradiation in a larva of *Temnopleurus reevesii*. The larva stays at the surface of seawater for a long time under dark conditions, but upon photoirradiation, it moves away from that position.
(MP4)

S3 Video The water current moving toward an individual larva (from the front) stopped and reversed direction after photoirradiation. This meant that the larva stopped swimming forward and began swimming backward.
(MOV)

S4 Video The water current before and after photoirradiation did not change in Opsin-2 morphants, suggesting that Opsin-2 is required for the response to photoirradiation. (MOV)
