# Supplementary figures and images for: Planktonic sea urchin larvae change their swimming direction in response to strong photoirradiation
Source: PLoS Genet. 2022 Feb 10;18(2):e1010033. doi: 10.1371/journal.pgen.1010033 (PMC8830728; doi:10.1371/journal.pgen.1010033)

*T. reevesii*   *H. pulcherrimus*

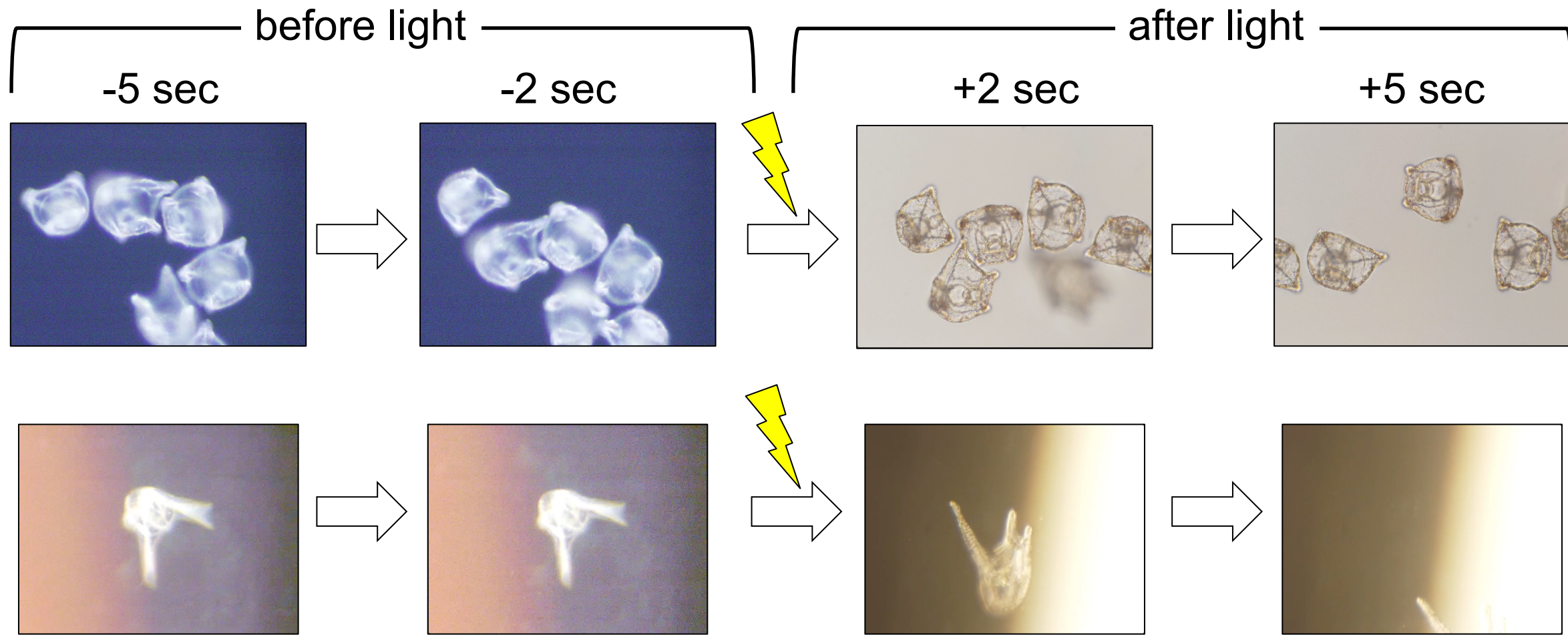

Supplement: S1 Fig — These are captured images from S1 and S2 Videos. The times (sec) shown on the images indicate the timing before and after photoirradiation. (PDF) [file pgen.1010033.s001.pdf]

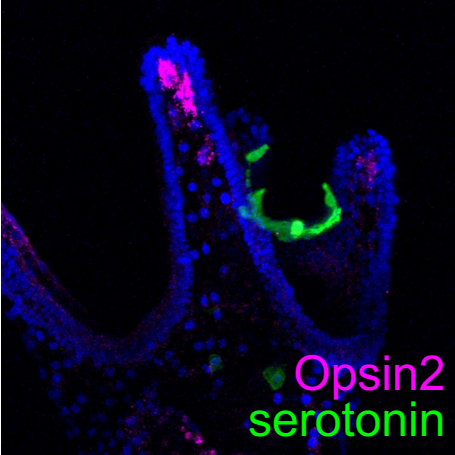

Random-MO

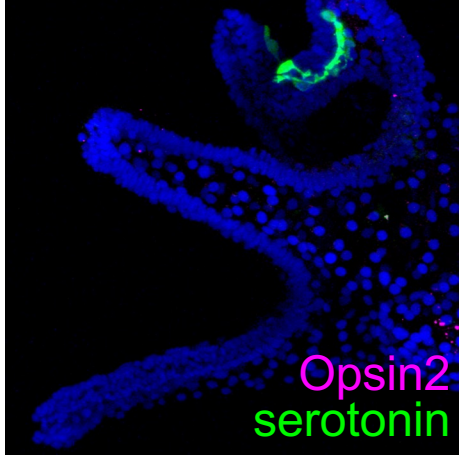

Op2-MO2

Supplement: S2 Fig — The Opsin2 protein was not detected in Opsin2-MO2 morphants. (PDF) [file pgen.1010033.s002.pdf]

A

ChAT

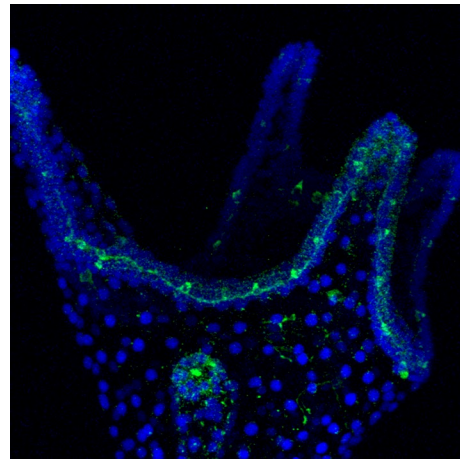

Random-MO

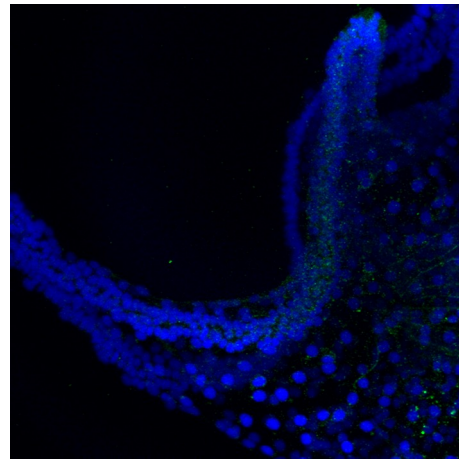

ChAT-MO1

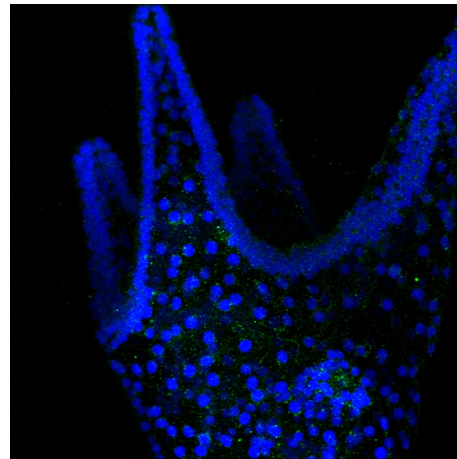

ChAT-MO2

B

rabbit anti-ChAT antibody

ChAT

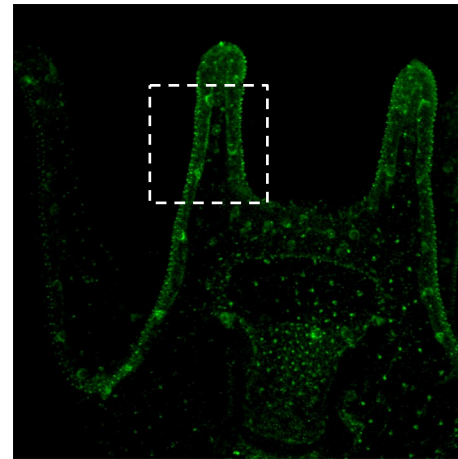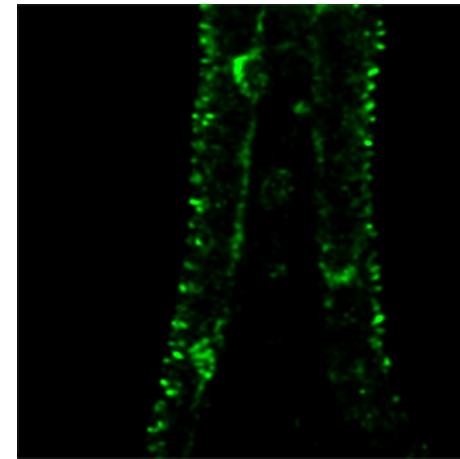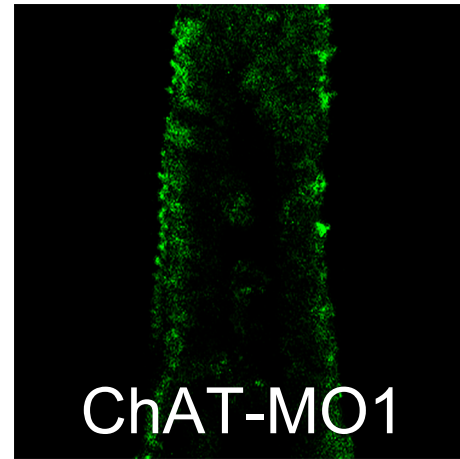

ChAT-MO1

preoral arm

Supplement: S3 Fig — (A) Two nonoverlapping morpholinos blocked the translation of ChAT. (B) Using the same antigen, we made an anti-ChAT antibody in a rabbit. Although the background was slightly high, the antibody recognized ciliary band neurons to a similar degree as a mouse antibody. The middle image is a magnified region of the dotted-line rectangle on the left. The right image shows the disappearance of the rabbit anti-ChAT antibody signal in the ChAT-MO1 morphant, supporting the specificity of the antibody. (PDF) [file pgen.1010033.s003.pdf]

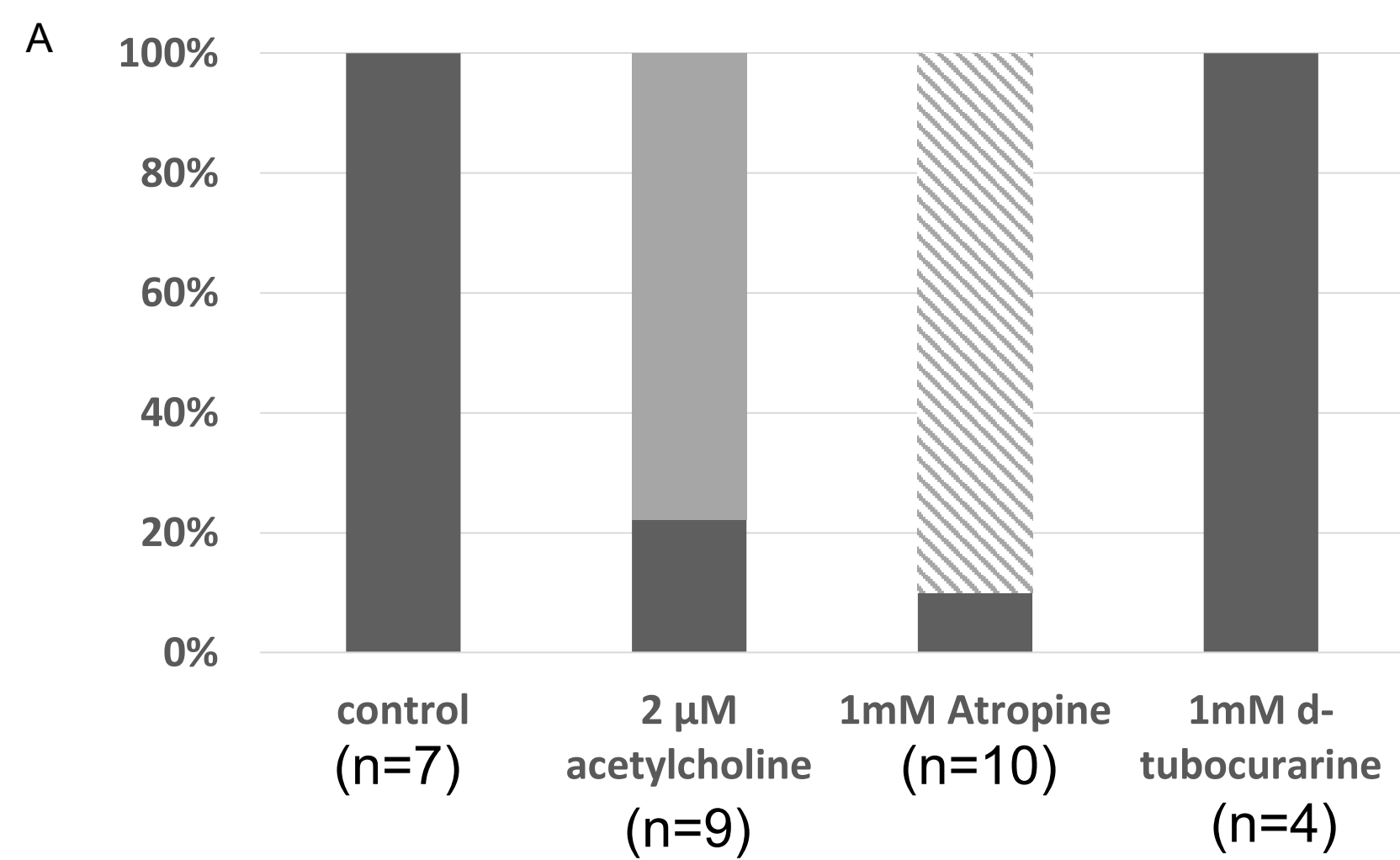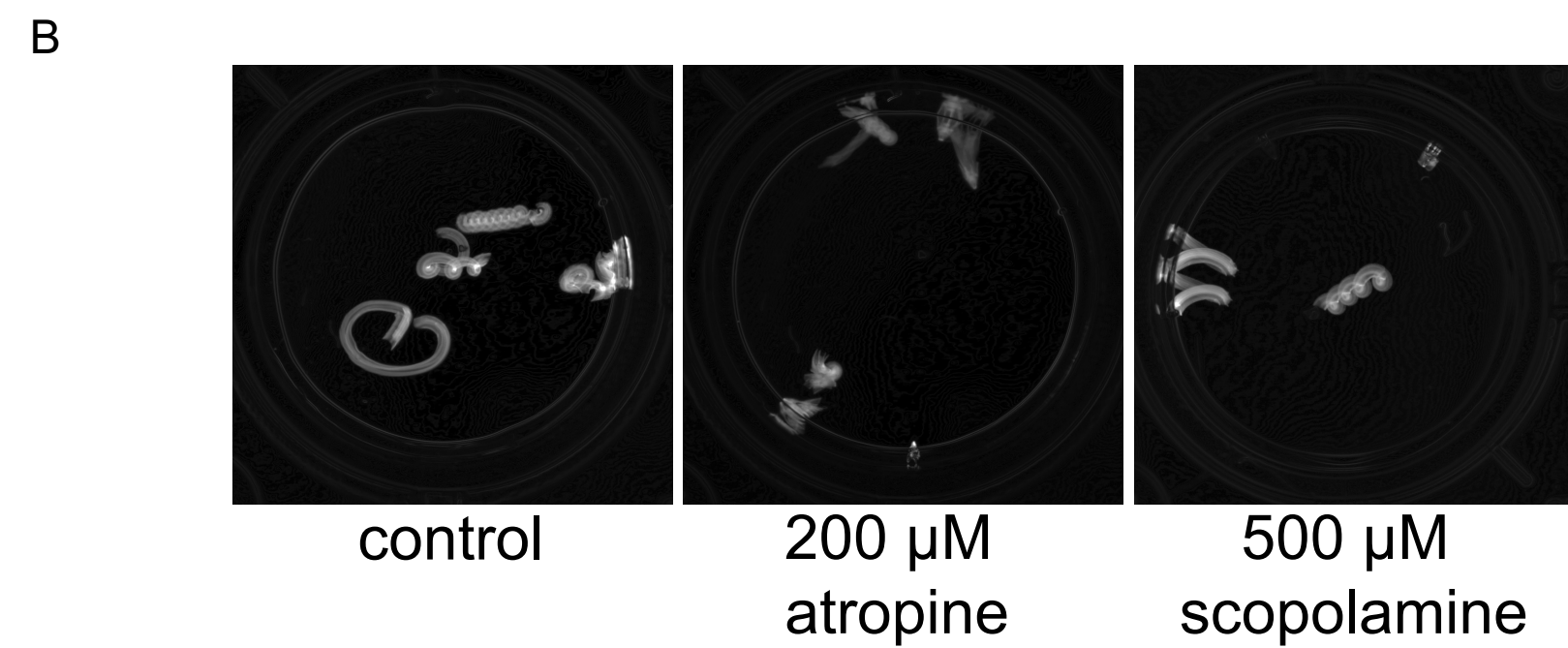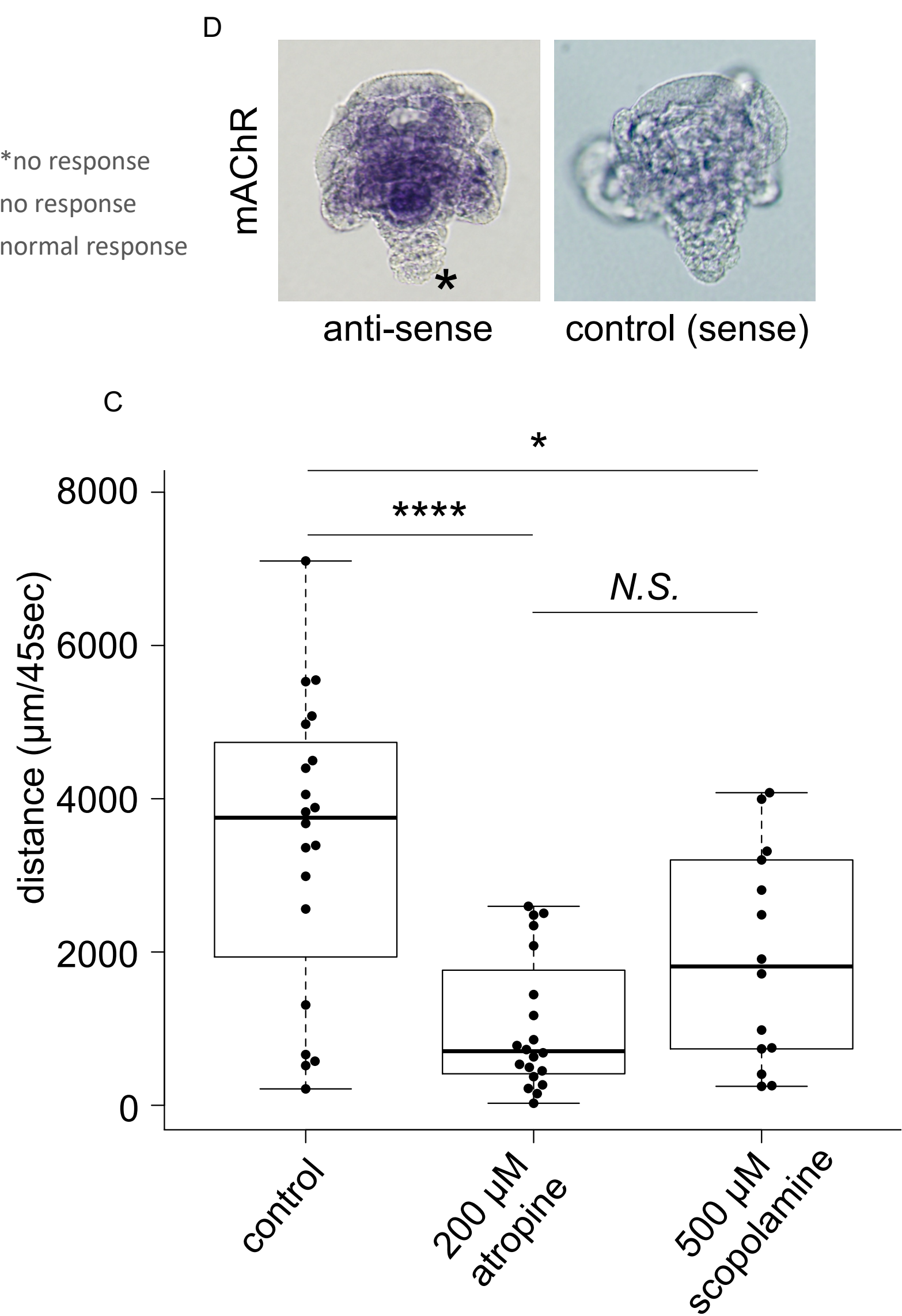

Supplement: S4 Fig — (A) The graph shows the change in particle direction before and after photoirradiation in the presence of acetylcholine and antagonists of its receptors. Excess acetylcholine inhibited the larval response to light. Because atropine-treated larvae basically did not show forward swimming, they did not respond to light. The nicotinic acetylcholine inhibitor d-tubocurarine did not inhibit the larval response to light input. (B) Superimposed images of 45 sec of swimming behaviors of sea urchin larvae (control [water-], atropine-, and scopolamine-treated). (C) The graph shows the distance of larval swimming shown in (C). Water-treated larvae (n = 20) could swim significantly longer distances than atropine-treated (n = 20) or scopolamine-treated (n = 14) larvae. *, p≤0.05, ****, p≤0.0001, N.S., not significant. (D) In situ hybridization for mAChR. mRNA of mAChR was expressed in the anterior regions of the larvae. The ciliary band region and posterior end (asterisks) had no strong expression. (PDF) [file pgen.1010033.s004.pdf]
